# Changes in Retinal Morphology, Electroretinogram and Visual Behavior after Transient Global Ischemia in Adult Rats

**DOI:** 10.1371/journal.pone.0065555

**Published:** 2013-06-11

**Authors:** Ying Zhao, Bo Yu, Yong-Hui Xiang, Xin-Jia Han, Ying Xu, Kwok-Fai So, An-Ding Xu, Yi-Wen Ruan

**Affiliations:** 1 Department of Central Nervous System Regeneration, Guangdong – Hongkong - Macau Institute of CNS Regeneration (GHMICR), Jinan University, Guangzhou, Guangdong, China; 2 Department of Neurology, the First Affiliated Hospital, Jinan University, Guangzhou, Guangdong, China; 3 Department of Human Anatomy, Jinan University School of Medicine, Guangzhou, Guangdong, China; 4 Department of Human Anatomy, Medical College, Shanghai University of Traditional Chinese Medicine, Shanghai, China; University of South Florida, United States of America

## Abstract

The retina is a light-sensitive tissue of the central nervous system that is vulnerable to ischemia. The pathological mechanism underlying retinal ischemic injury is not fully understood. The purpose of this study was to investigate structural and functional changes of different types of rat retinal neurons and visual behavior following transient global ischemia. Retinal ischemia was induced using a 4-vessel occlusion model. Compared with the normal group, the number of βIII-tubulin positive retinal ganglion cells and calretinin positive amacrine cells were reduced from 6 h to 48 h following ischemia. The number of recoverin positive cone bipolar cells transiently decreased at 6 h and 12 h after ischemia. However, the fluorescence intensity of rhodopsin positive rod cells and fluorescent peanut agglutinin positive cone cells did not change after reperfusion. An electroretinogram recording showed that the a-wave, b-wave, oscillatory potentials and the photopic negative response were completely lost during ischemia. The amplitudes of the a- and b-waves were partially recovered at 1 h after ischemia, and returned to the control level at 48 h after reperfusion. However, the amplitudes of oscillatory potentials and the photopic negative response were still reduced at 48 h following reperfusion. Visual behavior detection showed there was no significant change in the time spent in the dark chamber between the control and 48 h group, but the distance moved, mean velocity in the black and white chambers and intercompartmental crosses were reduced at 48 h after ischemia. These results indicate that transient global ischemia induces dysfunction of retinal ganglion cells and amacrine cells at molecular and ERG levels. However, transient global ischemia in a 17 minute duration does not appear to affect photoreceptors.

## Introduction

The retina is a light-sensitive tissue of the central nervous system. There are several types of cells in the retina; photoreceptors (rods and cones), located close to the outer surface of the retina, receive light stimulation and convert light into electrical signals; retinal ganglion cells (RGCs), near the inner retinal surface, transmit visual signals to the visual cortex and other higher visual centers; interneurons (bipolar cells and amacrine cells), located between the photoreceptors and ganglion cells, transmit signals from the photoreceptors to the RGCs [1].

Functional signals and activities of each cell type can be recorded by the electroretinogram (ERG). From an ERG, we can observe the following waves: the a-wave (produced by photoreceptors), the b-wave (conducted by the ON-bipolar cells) [2], the oscillatory potentials (OPs, triggered by amacrine cells) [3], and the photopic negative response (PhNR, induced by RGCs) [4]–[7].

The visual function of the retina can be also evaluated by different behavioral methods, such as startle reflex tests [8], orientation test [9], Y-maze for visual testing [10], and the optokinetic reflex [11]. These techniques have been used to detect either eye movement or navigation ability, which is affected by vision. Recently, visual discrimination apparatus of two types was developed to assess visual function in rats. One apparatus consisted of several compartments including a curved tube, one introduction chamber, two escape alleys, three swing doors and one home cage. This apparatus was designed to train rats to distinguish two different visual stimuli [12]. The other apparatus was the black-white box which is formed by two chambers (the white and black chamber) with a door opening between them. This was employed to test the white-black visual discrimination of rats [13]. If an animal perceives light, it will escape from the white chamber to the black chamber. Because the structure of this apparatus was adequate and simpler than the other apparatus mentioned above, it was employed in the present study to detect whether transient global ischemia affects light reception.

The model of transient global ischemia is a model in which the blood supply of the brain is completely blocked for a period of time. Clinically, transient global ischemia can occur during cardiac arrest, extracorporeal circulation, asphyxiation, and complex congenital heart lesions [14], [15]. During transient global ischemia, the blood supply of the retina is also completely blocked as it is derived from the internal carotid artery. It has been reported that transient global ischemia may cause sudden visual loss (amaurosis fugax) [16]. Therefore, to reveal the pathological mechanisms underlying transient global ischemia as it affects the retina will help us to understand how to protect retinal cells against ischemia.

In our previous research, we found that transient global ischemia, the 4-vessel occlusion (4-VO) model (permanent occlusion of vertebral arteries plus transient occlusion of carotid arteries), induced selective neuronal death and changes in dendritic and synaptic plasticity in the hippocampus [17]–[20]. However, we did not know whether retinal cells were also damaged by the same ischemic episode. In the present study we therefore used the same 4-VO model to investigate the impact of ischemia on the retina. In addition, we combined immunohistochemistry, ERG recording and visual behavior detection techniques to investigate how transient global ischemia affects different types of retinal cells and visual function.

The results showed that transient global ischemia induced a reduction in protein synthesis and the amplitudes of OPs and PhNR but did not block light perception. The structure and function of photoreceptors was also still intact.

## Materials and Methods

### Animals

Adult male Wistar rats (body weight 200–250 g) were used in the present study. All animal procedures were performed in strict accordance with the recommendations in the Guide for the Care and Use of Laboratory Animals of the National Institutes of Health. The protocol was approved by competent ethics committees at Jinan University. All efforts were made to minimize the suffering and number of animals used. Rats were randomly divided into five experimental groups: a control group (5 sham rats), Is (ischemia) 6 h group (5 rats sacrificed 6 h after ischemia), Is 12 h group (5 rats sacrificed 12 h after ischemia), Is 24 h group (5 rats sacrificed 24 h after ischemia) and Is 48 h group (5 rats sacrificed 48 h after ischemia). Ten rats were allocated for ERG recording. Twenty rats (10 rats in control group, and 10 rats in Is 48 h group) were employed for visual behavior detection.

### Transient Global Ischemia

Transient global ischemia was induced using the 4-VO method [21] with modifications (detection of ischemic depolarization) [22]. Briefly, animals were fasted overnight (8–12 h) to produce uniform blood glucose levels. For surgery, animals were anesthetized with 10% chloral hydrate (0.4 ml/100 g; 27500, HaoMa, Guangzhou, China) by intraperitoneal injection. A silicone tube was placed loosely around each carotid artery to allow subsequent occlusion of the vessels. The animals were then placed in a stereotaxic frame and the bilateral vertebral arteries were electrocauterized. A microelectrode filled with 2M KCl was inserted into the hippocampus (2.5 mm below the brain surface) to record ischemic depolarization (ID) with an amplifier (Neuroprobe 1600, A-M System, Carlsberg, MA, USA). ID is defined as the direct current potential (DCP) from an amplifier shifted from zero to approximately −20 mV after ischemia. The duration of ID was determined by measuring the period from the beginning of the DCP reaching −20 mV to the point where the potential started at 0 mV (repolarization) after reperfusion. Transient global ischemia was produced by occluding both common carotid arteries for 17.33±0.76 min to induce ID for 12.21±0.36 min. Animals were reperfused by releasing the occlusion of bilateral common carotid arteries. Rats survived for 6 h, 12 h, 24 h or 48 h after reperfusion.

### Tissue Processing

Rats were anaesthetized with 10% chloralhydrate (0.4 ml/100 g). Perfusion-fixation was carried out transcardially with 0.9% normal saline (NS) followed by 4% paraformaldehye in phosphate buffer (PB, 0.15M, PH 7.4). Eyeballs were immediately removed and post-fixed in the same fixative for 2 days. Eye tissues were processed with a series of ascending alcohols; cleared in trichloromethane; then embedded in paraffin. Retina paraffin blocks were cut horizontally at 7 µm thickness using a microtome (RM2235, Leica, Wetzlar, Germany). Six nonadjacent sections through the optic disc with an interval of 70 µm were selected from each rat, and two were mounted on a glass slide coated with gelatin. Each slide contained 10 sections from five experimental groups to ensure all group sections were processed under the same conditions.

### Histochemistry

Sections for hematoxylin and eosin (HE) staining were first de-waxed in xylene, rehydrated in a series of descending alcohols, rinsed in deionized distilled water (DDW), and stained with HE. Stained sections were dehydrated in a series of ascending alcohols, cleared in xylene and mounted with coverslips.

Sections for fluorescent peanut agglutinin (PNA) staining were processed based on Blanks’ study [23]. Briefly, sections were de-waxed and rehydrated as described above, then rinsed in phosphate buffered saline (PBS, 0.01M, PH 7.4) followed by incubation in 0.01M PBS containing 0.1% Tween-20 for 30 min at room temperature. The sections were transferred into 50 µg/ml fluorescent-conjugated PNA (FL-1071, Vector Laboratories, Burlingame, USA) in PBS-Tween for 1 h at room temperature, then rinsed in PBS-Tween and 0.01M PBS. The sections were mounted on glass slides with coverslips coated with an anti-quenching reagent and observed with a Leica epifluorescence microscope (DM6000B, Leica, Wetzlar, Germany).

### Immunofluorescence

Sections for immunofluorescence were first de-waxed in xylene and rehydrated as described above, then rinsed in 0.01M PBS. To expose the antigens, sections were placed in citrate buffer in a water bath kettle and heated to 90°C for 15 min. After cooling to room temperature, the sections were incubated in blocking solution (5% normal goat serum, 1% BSA and 0.25% Triton X-100 in 0.01M PBS) for 1 h at room temperature, then incubated with primary antibody diluted in blocking solution overnight at 4°C. The names and concentrations of the four primary antibodies are listed in Table 1. After rinsing in 0.01M PBS, the sections were incubated in fluorescent secondary antibodies at room temperature for 1 h (Table 1) followed by washes with 0.01M PBS. Afterwards, the sections were mounted with anti-quenching reagent and a coverslip. Finally the labeled tissues were observed and photographs taken using a fluorescence microscope (DM6000B, Leica, Wetzlar, Germany) with the appropriate filters.

**Table 1:** Information on primary and secondary antibodies.

Primary antibodies	Host	Dilution	Catalogue	Source
β3-Tubulin(TU-20)	Mouse	1∶1000	4466	Cell Signaling
Calretinin	Rabbit	1∶4000	AB5054	Millipore
Recoverin	Rabbit	1∶1000	AB5585	Millipore
Rhodopsin	Mouse	1∶1000	MAB5356	Millipore
**Secondary antibodies**				
Alexa Fluor 488	Goat anti-mouse	1∶500	115-545-003	Europe Ltd
Alexa Fluor 488	Goat anti-rabbit	1∶500	115-545-003	Europe Ltd

### Cell Counts

Based on HE staining and immunofluorescence of βIII-tubulin, calretinin and recoverin, cells were quantified in the retinal ganglion cell layer (GCL) and/or inner nuclear layer (INL). Six sections of retina, through the optic disc, were selected from each rat. Two photographs were taken per section 100 µm either side of the optic disc, with a 40x objective. Because there were 5 rats in each experimental group, a total of 60 images from 30 sections were quantitatively analyzed from each group. The number of cells in a 300 µm length of the GCL and/or INL was counted from the captured images using the cell counter of the Image J software program.

### Cell Layers and Thickness

Due to the condensed arrangement of cells in the inner nuclear layer (INL) and outer nuclear layer (ONL), it was difficult to quantify cells accurately. The INL and ONL in HE stained sections were analyzed by counting cell layers and measuring the thickness of the INL and ONL. The number of sections and magnification were the same as that used for cell counting. The numbers of cell layers in the INL and ONL of HE stained sections were counted directly from the captured images. The thickness of the INL and ONL were measured using Adobe Photoshop CS6 software.

### Fluorescence Intensity

To detect rhodopsin immunoreactivity and fluorescent PNA staining, images were captured under the same conditions, including light exposure time and magnification. The number of captured images was the same as that used for cell quantification. Fluorescence intensity in each experimental group was analyzed with Image J software.

### Electroretinography (ERG)

Rats should usually be adapted to the dark prior to ERG recordings. Based on Wang et al [24], the time for dark-adaptation in the present study was at least 70 min to reach a stable status. Following stabilization, ERGs were obtained. In brief, the control rats were anesthetized with 10% chloralhydrate (0.4 ml/100 g) and the pupils dilated with tropicamide. Recording electrodes were placed on the corneas. Two reference electrodes were inserted into subcutaneous tissue behind the ears and one ground electrode was inserted into subcutaneous tissue of the tail. The a-wave, b-wave, OPs and PhNR were recorded using a Roland Consult electrophysiological diagnostic system (Brandenburg, Germany).

Scotopic ERGs (a-wave, b-wave, OPs; 3.0 cds/m^2^, white flash) were first recorded after 70 min dark adaptation. After light adaption under a continuous blue background (25 cds/m^2^) for 5 min to suppress rod cell electrical activity, PhNR was recorded with red flashes (3.0 cds/m^2^). Ischemic operation was performed on the control rats after ERG recording. After electrocauterizing the vertebral arteries and isolating the common carotid arteries under lighted conditions, rats were sent to a dark-adaptation room for 70 min, followed by transient occlusion of the common carotid arteries by arterial clamps (beak 8 mm) for 17.30±0.30 min. The rats were evaluated using ERG during ischemia, 1 h and 48 h after ischemia.

**Figure 1:**
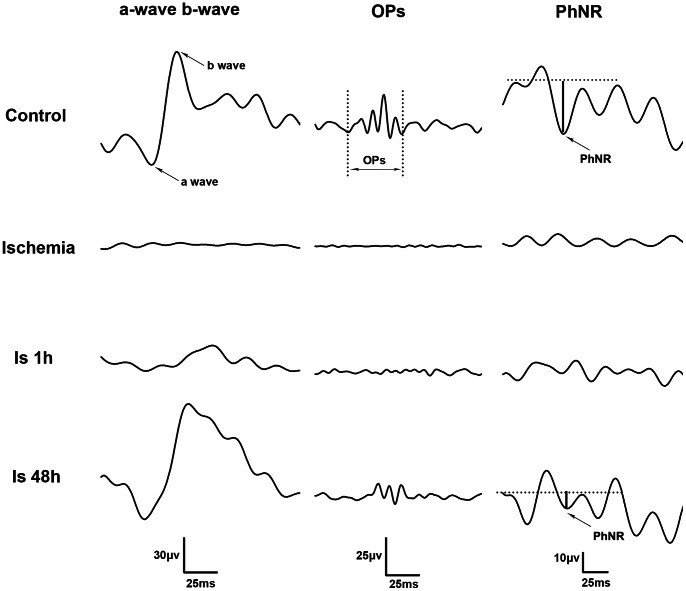
Different waves in ERG recordings before and after ischemia. Representative dark-adapted ERG component amplitudes (a-wave, b-wave and OPs) and amplitudes of PhNR were recorded before and during ischemia, 1 h and 48 h after ischemia.

The measurement of different waves is illustrated in Figure 1. The amplitude of the a-wave was measured from the baseline to the peak of the a-wave, and the amplitude of the b-wave was measured from the trough of the a-wave to the peak of the b-wave. The amplitude of OPs was determined as the sum of the amplitudes of OP1, OP2, OP3, and OP4. The amplitude of PhNR was defined as the length from baseline to the first trough immediately following the b-wave. Four waves were measured using pCLAMP9.2.

### Visual Behavior Detection

**Figure 2:**
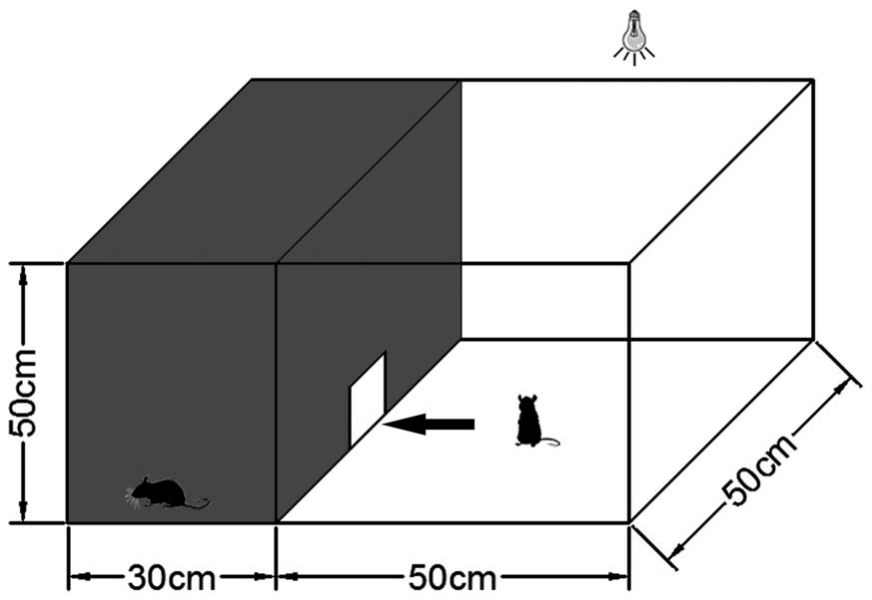
Schematic diagram illustrating the black-white box. The rat was placed in the middle of the white compartment at the start of the trial, and could travel freely in the white and black chambers through an aperture between the chambers.

Visual behavior detection was conducted using a black-white box (custom-made by Metronet Technology Ltd). There were two chambers in the box: the black chamber and white chamber (Fig. 2). An aperture (10 × 12 cm) between the black and white chambers allowed rats to travel freely from one to the other. The black chamber was illuminated with infrared light, while the white chamber was illuminated by bright white light. Two cameras, installed in the two chambers separately, captured activities of the rats and were connected to a Noldus EthoVision XT 8.0 recorder and monitor.

Rats were placed into the middle of the white compartment at the start of the trial and left in the box for 15 min. The number of intercompartmental crosses, distance moved, mean velocity and the time spent in the black chamber and white chamber were recorded by the Noldus EthoVision XT 8.0 software.

### Statistical Analysis

All data were analyzed with the statistical software program SPSS17.0 and were presented as mean ± SEM. One-way Analysis-of-variance (ANOVA) followed by the SNK (Student-Newman-Keuls) multiple comparison tests was used. *P*-values <0.05 were considered as significant.

## Results

### No Changes in Cell Numbers of the GCL, INL and ONL after Ischemia

**Figure 3:**
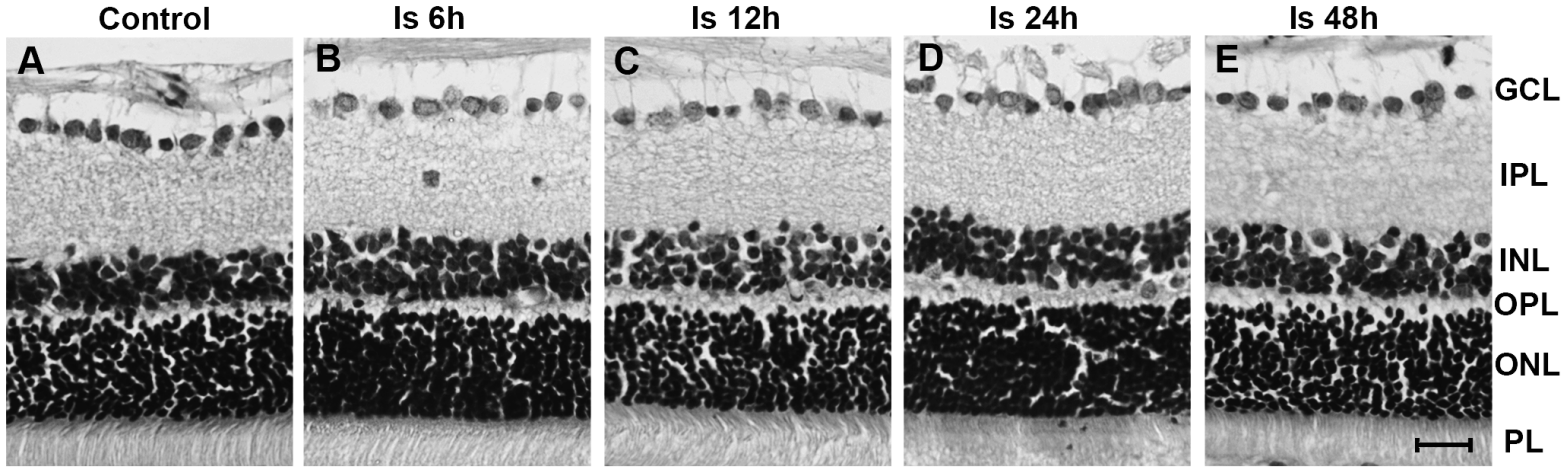
Photomicrograph showing HE staining of cell layers in the retina. Several layers of cells were identified from outside to inside of the retina as follows: the photoreceptor layer (PL), outer nuclear layer (ONL), outer plexiform layer (OPL), inner nuclear layer (INL), inner plexiform layer (IPL) and the ganglion cell layer (GCL). Cell bodies were seen in the GCL, INL and ONL. There were no obvious morphological changes in different groups before and after ischemia under a light microscope (A-E). Scale bar: 20 µm.

Cell layers of the retina were identified using HE staining (Fig. 3). No obvious changes in the morphology of cells in the GCL, INL and ONL of the retina were observed after ischemia. Quantitative analysis also showed no significant differences in the number of RGCs in the GCL, or the thickness and number of cell rows in the INL and ONL between the control group and ischemic groups, all *p*>0.05 (Table 2).

**Table 2:** Changes in the number, thickness and cell layers of the retina.

Groups	n	Number of cells in GCL	Thickness of INL (µm)	Number of rows in INL	Thickness of ONL (µm)	Number of rows in ONL
Control	5	27.66±0.57	18.78±0.42	4.58±0.07	34.41±0.61	9.73±0.20
Is 6h	5	26.16±0.54	19.00±0.35	4.23±0.06	33.47±0.60	9.19±0.17
Is 12h	5	26.74±0.39	20.10±0.53	4.32±0.06	33.66±0.68	9.46±0.17
Is 24h	5	27.60±0.51	18.54±0.62	4.30±0.06	32.17±0.88	8.84±0.18
Is 48h	5	27.89±0.47	18.40±0.34	4.17±0.05	32.54±0.51	9.04±0.17
		*P*>0.05	*P*>0.05	*P*>0.05	*P*>0.05	*P*>0.05

All groups were compared with the control group.

### Reduction of βIII-tubulin, Calretinin and Recoverin Immunoreativities after Ischemia

**Figure 4:**
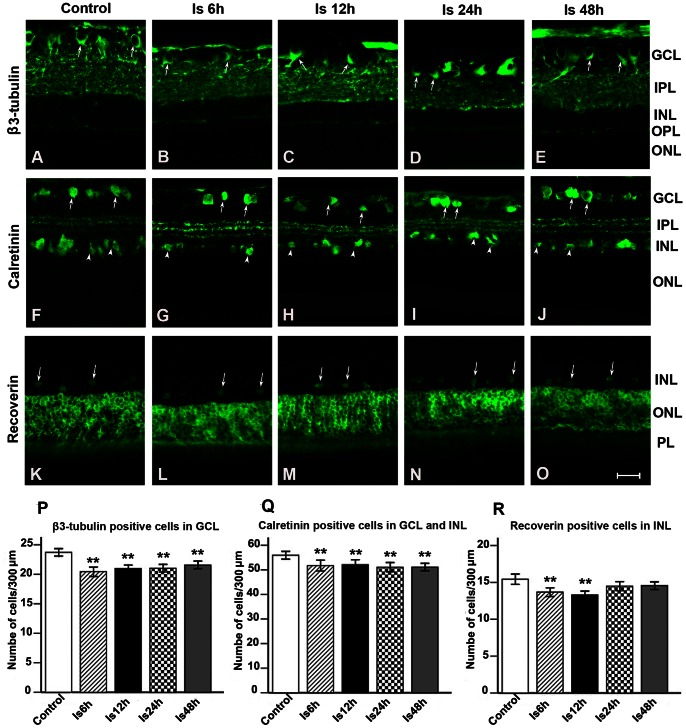
Immunofluorescent reactivities and cell numbers in different retinal layers. A-E: βIII-tubulin immunoreativity was observed in the somata of retinal ganglion cells (RGCs) in the ganglion cell layer (GCL) (arrows, A-E) and processes in the inner plexiform layer (IPL). F-J: Calretinin positive cells were found in the GCL (arrows, F-J) and INL (arrow heads, F-J) and three positive strata of processes appeared in the IPL. K-O: Recoverin immunoreactivity was detected in retinal photoreceptors and cone bipolar cells in the inner nuclear layer (INL) (arrows, K-O). Quantitative analysis showed that the number of βIII-tubulin positive cells in the GCL decreased from 6 h to 48 h after ischemia, *p*<0.01(P). The number of calretinin positive amacrine cells in the GCL and INL also decreased from 6 h to 48 h after ischemia, *p*<0.01 (Q). The number of recoverin positive cells in the INL was reduced at 6 h and 12 h after ischemia, *p*<0.01, but returned to control levels at 24 h after reperfusion, *p*>0.05 (R). Scale bar: 20 µm.

RGCs were identified using anti-βIII-tubulin immunostaining. βIII-tubulin positive cell bodies of RGCs in the GCL, and positive dendrites of RGCs in the INL were identified (Fig.4 A-E). Calretinin labeling appeared in amacrine cells of the GCL and the innermost layer of the INL. Three layers of dendrites of calretinin positive cells appeared in the IPL (Fig.4 F-J). Recoverin immunoreactivity was detected in photoreceptors of the ONL and cone bipolar cells in the INL (Fig.4 K-O).

Qualitative analysis showed that the number of βIII-tubulin positive cells in the 300 µm length of the GCL was 23.72±0.31 in the control group. This number was significantly decreased in the Is 6 h (20.44±0.40), Is 12 h (20.96±0.30), Is 24 h (21.02±0.33) and Is 48 h (21.55±0.33) groups when compared with the control level, *p*<0.01(Fig. 4P). Similarly, the number of calretinin positive cells in the GCL and INL was also decreased in the Is 6 h (51.71±1.10), Is 12 h (52.11±1.02), Is 24 h (51.06±0.94) and Is 48 h (51.09±0.77) groups in comparison to the control group (55.94±0.81), *p*<0.01 (Fig. 4Q). However, the alteration patterns in recoverin immunoreactivity of the INL were slightly different from βIII-tubulin and calretinin immunoreactivities after ischemia. The number of recoverin positive cells in the control group was 15.43±0.36, but it reduced to 13.69±0.29 at Is 6 h and 13.29±0.27 at Is 12 h after ischemia, *p*<0.01. The number of recoverin positive cells then returned to the control level at Is 24 h (14.49±0.31) and Is 48 h (14.57±0.26), *p*>0.05 (Fig. 4R).

### No Alteration of Rhodopsin Immunoreactivity and Fluorescent PNA Intensity after Ischemia

**Figure 5:**
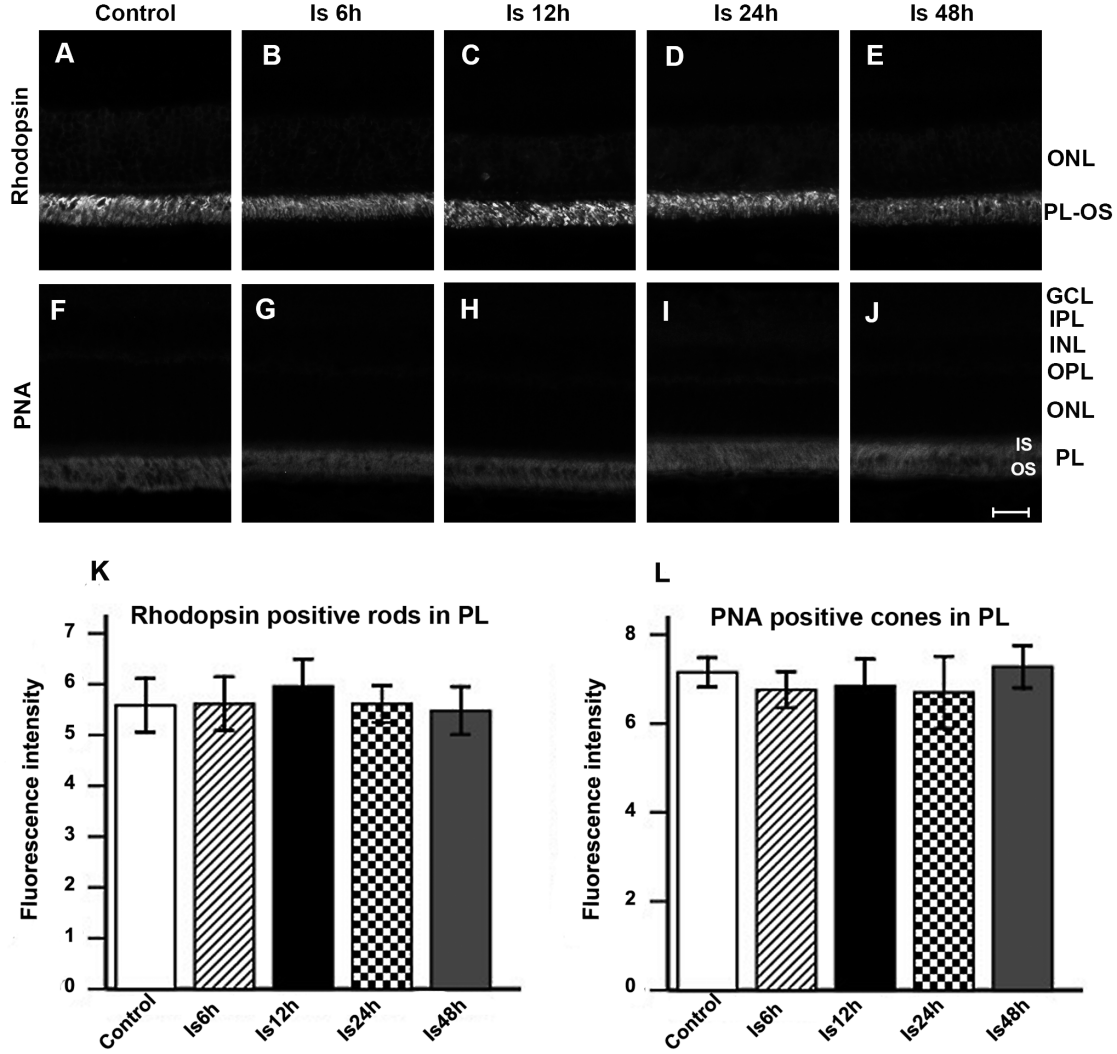
Rhodopsin fluorescent immunoreactivity and PNA fluorescent intensity. A-E: Rhodopsin positive cells (specific for rods) appeared in the outer segment of the photoreceptor layer of retina (PL-OS). F-J: PNA positive cells (specific for cones) were found in the inner segment (IS) and the outer segment (OS) of the photoreceptor layer (PL). Quantitative analysis showed that the fluorescence intensity of rhodopsin positive cells in the PL did not change after ischemia, *p*>0.05 (K). The fluorescence intensity of PNA positive cells also did not change after ischemia, *p*>0.05 (L). Scale bar: 20 µm.

Rhodopsin was strongly expressed in the outer segment layer (PL-OS) of the retina (Fig.5 A-E). The inner segment (IS) and outer segment (OS) of cones were specifically labeled with fluorescent PNA staining in the photoreceptor layer of the retina (Fig.5 F-J). Quantitative analysis showed that there were no significant differences in either rhodopsin fluorescent intensity or PNA fluorescent intensity before and after ischemia, *p*>0.05 (Fig.5 K&L).

### Different Wave Changes in ERG Recordings after Ischemia

ERG recordings were performed before ischemia, during ischemia, 1 h and 48 h after ischemia on the same rats. From figure 1, amplitudes of the a-wave, b-wave, Ops and PhNR were completely lost during ischemia. From Fig1 and Table 3, the amplitudes of the a- and b-waves at 1 h were still reduced after reperfusion when compared with the control group, *p*<0.05. The amplitudes of the a-wave and the b-wave almost recovered to the control level at 48 h after reperfusion, *p*>0.05. Although there was no significant difference between the control group and the ischemic groups, the values of the b/a-wave ratio at 1 h and 48 h after ischemia were only half of the control level (Table 3). The amplitudes of OPs and PhNR still disappeared during and 1 h after ischemia. Although they were present 48 h after reperfusion, the OPs and PhNR amplitudes were still smaller than the control level, *p*<0.01 (Fig.1 and Table 3).

**Table 3:** Changes in ERG recordings before and after ischemia.

Groups	n	a-wave (µV)	b-wave (µV)	b/a-wave ratio	Ops (µV)	PhNR (µV)
Control	10	28.68±2.76	52.50±4.93	2.77±1.02	42.92±2.84	24.50±3.07
Ischemia	10	disappeared	disappeared	disappeared	disappeared	disappeared
Is 1h	10	18.56±3.55*	16.25±1.31**	1.49±0.40	disappeared	disappeared
Is 48h	10	32.55±2.99	44.35±3.45	1.46±0.17	28.91±2.45**	10.40±1.84**

* _:_ P<0.05, **: P<0.01, compared with the control group.

### Alteration of Visual Behavioral Detection after Ischemia

**Figure 6:**
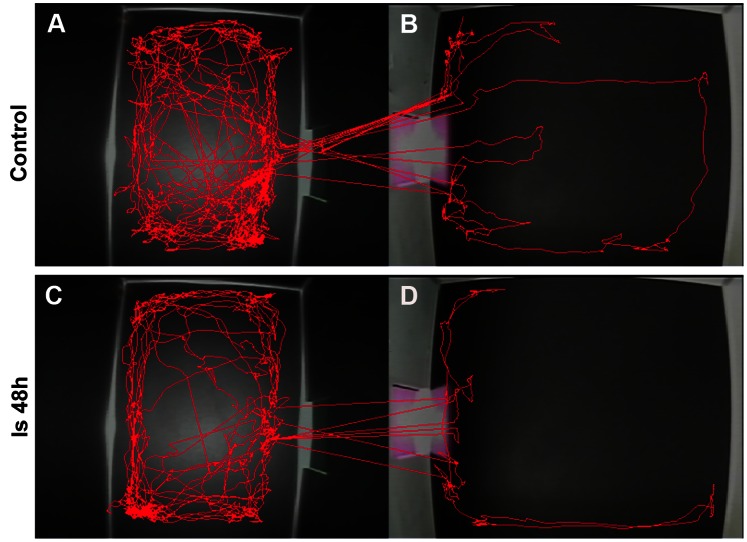
Behavioral detections of rats in the black-white box. The red lines represent the moving trajectory of a single rat in the two experimental groups. The representative rat in the control group spent more time traveling in the black chamber (A) than in the white chamber (B). The ischemic rat traveled in the black-white box in a similar pattern as that in the control group (C & D).

**Figure 7:**
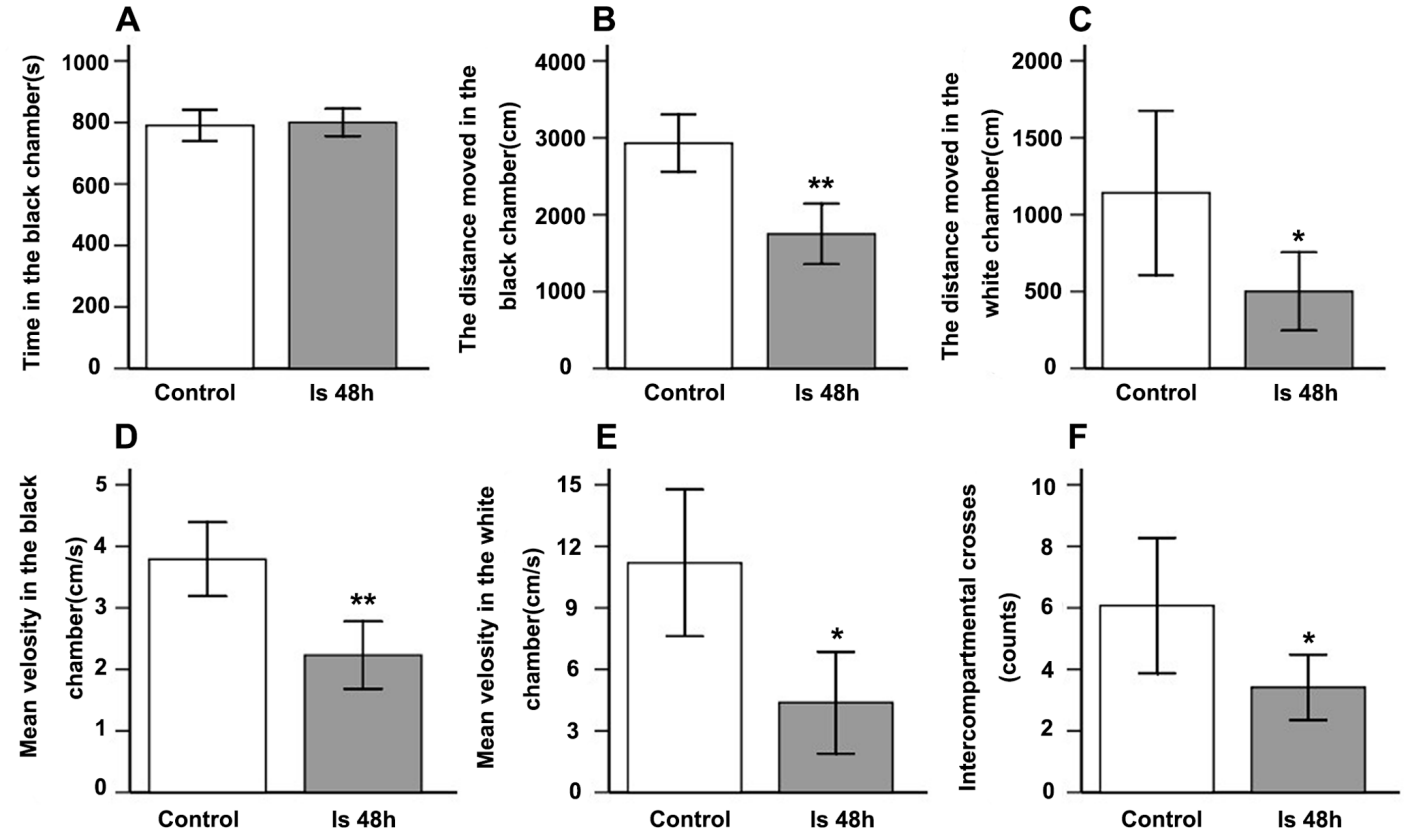
Statistical analysis of the parameters of behavior detection. There was no difference in the time spent in the black chamber between the control and the ischemic groups at 48 h after reperfusion,*p*>0.05 (A). However, the distance moved in the black chamber (B, *p*<0.01) and white chamber (C, *p*<0.05), the mean velocity in the black (D, *p*<0.01) and white chamber (E, *p*<0.05), and the number of intercompartmental crosses (F, *p*<0.05) were reduced at 48 h after reperfusion.

Rats prefer staying in dark places. Therefore, when a rat was placed in the center of the white chamber (Fig.2 and Fig.6 B&D), it would enter the black chamber through an aperture between the white and black chamber. Rats stayed in the dark chamber for a longer time than in the white chamber in both the control group (Fig.6 A&B) and ischemic group (Fig.6 C&D). Statistically, there was no significant difference in the duration in the dark chamber between the control group (790.19±23.80 s) and Is 48 h group (800.15±12.37 s, *p*>0.05 (Fig.7 A). However, the distance moved in the black chamber was reduced in the Is 48 h group (1750.16±180.87 cm) when compared with the control group (2930.49±171.17 cm), *p*<0.01 (Fig.7 B); and in the white chamber was decreased in the Is 48 h group (500.42±115.74 cm) when compared with the control group (1141.92±242.67 cm), *p*<0.05 (Fig.7 C). Mean velocity at 48 h after ischemia in black chamber (2.23±0.25 cm/s) were also reduced when compared to control animals (3.79±0.28 cm/s), *p*<0.01 (Fig.7 D); and in white chamber was decreased in the Is 48 h group (4.38±0.78 cm/s) when compared with the control group (11.19±1.63 cm/s), *p*<0.05 (Fig.7 E). Finally, the number of intercompartmental crosses in the control group was 6.07±1.02/900 s, whereas it decreased at 48 h after ischemia (3.42±0.48/900 s), *p*<0.05 (Fig.7 F).

## Discussion

In the present study, we investigated changes in different types of neurons of the retina using a combination of immunohistochemistry, ERG and visual behavior detection following transient global ischemia. The results revealed varied vulnerability among different retina cell types to ischemia.

### RGCs and Amacrine Cells are the Most Sensitive to Ischemia in the Retina

The severity of ischemic effects on RGCs depends on the degree and duration of ischemia. In the present study, we employed a transient global ischemia model (4-VO) without mechanical injury. Retinal ischemia was induced by permanent occlusion of the vertebral arteries plus transient occlusion of the bilateral common carotid arteries for approximately 17 min. Although we did not detect changes in the number of individual cell layers after ischemia, we found that βIII-tubulin immunoreactivity (RGCs marker) decreased from 6 h to 48 h after reperfusion. This suggests the function of RGCs was affected by the transient ischemic attack. Consistent with this, the PhNR wave was completely lost during ischemia and 1 h after reperfusion. Even at 48 h after reperfusion, the amplitude of PhNR was still not recovered, which indicated that the function of RGCs had not completely recovered by this time. ERG has been considered a more sensitive method to detect retinal injury than histology [25]. The amplitude of PhNR was considered a marker to reflect the activity of RGCs [4]–[7]. Based on previous studies, the amplitude of PhNR was reduced in glaucoma [6], retinal vein occlusion [7], severe diabetic retinopathy [5] and after optic nerve transection [4]. Compared to transient global ischemia, the intraocular pressure (IOP) model produced much more severe ischemic and mechanical injury to RGCs. When the IOP was elevated to 90 to 120 mmHg for 45 to 90 min, the majority of RGCs died by 7 days or later after reperfusion [26], [27]. A similar phenomenon was observed following ischemia caused by ligature of the ophthalmic vessels (LOV) [28]. Although internal carotid artery occlusion (ICAO) and occlusion of bilateral common carotid arteries (BCCAO) induced milder damage to RGCs than that induced by IOP or LOV, a longer ischemic episode (2 h in ICAO, permanent in BCCAO) also caused death of most RGCs at different stages following reperfusion [29]–[31].

Therefore, previous morphological studies indicated that RGCs die after severe ischemia. In the present study, morphological and ERG recording techniques showed that the function of RGCs was affected even though no changes in cell number under transient global ischemia attack were observed, which suggests RGCs are vulnerable to ischemia.

In the retina, the amacrine cell is also sensitive to ischemia. It has been reported that 30% calretinin positive amacrine cells were lost after 60 min of ischemia by elevating intraocular pressure to 100–120 mmHg, or by occlusion of the middle cerebral artery at 1–3 days reperfusion time [32]. After ligation of the internal carotid artery for 2 h, almost half of calretinin positive amacrine cells were lost 22 h following reperfusion [31]. In Dijk, et al’ s study, approximately 80% calretinin positive amacrine cell loss was observed at 3 days after 60 min ischemia in the IOP model [33], [34]. However, Hernandez et al’s study showed that calretinin immunoreactivity of amacrine cells recovered at 7 days after reperfusion [35].

Compared with the above studies, amacrine cells underwent similar changes as RGCs in immunoreactivity after 17 min transient global ischemia in the present study. Approximately 10% of calretinin immunoreactive cell loss occurred from 6 h to 48 h after transient global ischemia.

In ERG studies, OPs are mainly generated by amacrine cells. It has been reported that amplitudes of OPs reduced after elevating IOP [27]. Our study has shown that transient global ischemia for 17 min completely blocked OPs during ischemia and 1 h after reperfusion. The amplitudes of OPs were still lower than the control level at 48 h after ischemia. This evidence suggests that amacrine cells are also sensitive to different types of ischemic injuries.

### Bipolar Cells and Photoreceptors are Tolerant of Ischemia

Morphologically, we did not find changes in cellular layers, the thickness of the ONL, rhodopsin immunoreactivity (rods marker) or PNA fluorescent density (cones marker) before and after ischemia in the present study. These results indicate photoreceptors are most tolerant to ischemic insult. In support of this, it has been reported that the number of horizontal cells and photoreceptors of the rat retina did not change while the majority of RGCs and several subtypes of amacrine cells were lost in glaucoma models [34], [36], [37]. Essentially, the sensitivity to ischemia of different cell types depends on the degree and duration of ischemia. When ischemic duration is extensive, such as permanent ischemia, photoreceptor cells will eventually degenerate. Stevens and his colleagues reported that photoreceptors were lost at 3 months following permanent ligation of the bilateral common carotid artery [29].

Based on the results of ERG recordings in the present study, the a-wave disappeared during ischemia, however, it partially recovered at 1 h and completely recovered at 48 h after reperfusion. These electrophysiological results further indicate that photoreceptor cells can quickly recover after reperfusion.

Although we did not find changes in cellular layers or the thickness of the INL, the number of recoverin positive cells (cone bipolar cells) of the INL was reduced at 6 h and 12 h after reperfusion. However, this number returned to the control level 24 h after ischemia. ERG recording results from our study have also showed that the b-wave (produced by bipolar cells) disappeared during ischemia and was barely seen at 1 h following reperfusion, but it recovered by 48 h after reperfusion. The changing pattern of the b-wave after ischemia was similar to that of the a-wave, but the b/a-wave ratio decreased to almost half of the control value at 1 h and 48 h following ischemia. The b/a-wave ratio has been considered a sensitive parameter for detecting the degree of retinal injury induced by ischemia. Lower b/a-wave ratios were considered as indicators of severe injury of the retina [38], [39]. It has been reported that the b-wave was totally abolished while a-wave amplitude enhanced without any histological changes of retinal neurons at day 7 following BCCAO [40]. Therefore, the results of morphology and ERG recordings in our study indicate that the function of bipolar cells was transiently affected by ischemia.

Our study has shown that RGCs and amacrine cells are most vulnerable to ischemia, while photoreceptors are most tolerant, and bipolar cells are in between. These results are consistent with those from Yamamoto and his colleagues [41]. They investigated the order of neuronal death in the retina using a BCCAO model and found that RGCs were damaged at 1 week after ischemia while INL neurons were dead at 2 months; photoreceptors were absent at 4 months after permanent BCCAO.

### Ischemic Changes in Visual Behavior Detection

Although the black-white box was widely used to detect anxiety by stress [42]–[45], it has recently been employed to investigate visual function [13]. An animal with light perception will escape from the white chamber to the black chamber. Based on Lin’s report, normal mice stayed in the black chamber for a longer time, whereas retinal degeneration *rd/rd* mice stayed in the black chamber for a shorter time because night-sensitive photoreceptors (rod cells) have been damaged. In the present study, there was no significant difference in the duration of remaining in the black chamber between the control group and ischemic group, which indicates that the animals still perceive light. However, ischemic rats showed a reduction in the distance moved and mean velocity in the black and white chamber, which may reflect a week visual acuity. In support of this, ERG results showed that the amplitudes of OPs and PhNR were reduced after ischemia. In addition, the protein synthesis of ganglion cells and amacrine cells was decreased within 2 days after ischemia in spite of an unchanging number of these cells. Therefore, transient global ischemia in a period of 17 min not only induces dysfunction in protein synthesis and electrophysiology but also in visual behavior.

Rats have a rod-dominated retina and prefer dark places where they feel more comfortable [46]. This is different with the human who is largely dependent upon cone-mediated vision. Therefore, the criteria for evaluating anxiety are different between the human and the rat. Based on previous studies, the criteria for evaluating anxiety behavior in rats includes the time within the black chamber and white chamber, the number of intercompartmental crosses, and activity in the white chamber and black chamber [42]–[44], [47], [48]. In the present study, we also explored whether transient global ischemia would induce anxiety behavior. We found that the number of intercompartmental crosses decreased in ischemic rats compared with controls, suggesting that anxiety-like behavior appears in rats after transient global ischemia. Based on Luan et al’s finding that alpha/Y-like retinal ganglion cells innervate the midbrain dorsal raphe nucleus [49] and ablation of this type of RGC induced depressive-like behavior [50], it is possible that transient global ischemia induces this type of cell injury thus causing anxiety-like behavior. This behavior may indeed be due to changes in the retina that causes visual impairment but we cannot discount the possibility that anxiety-like behavior is also caused by changes to brain regions affected in this model of ischemia.

### Impacts of Different Ischemia Models on the Pathology of the Retina

Different ischemic retina models were employed to investigate the impact of ischemia on the retina. A model of high IOP was used to investigate ischemic retinal autophagy [51], [52]. The model by ligation of the optic nerve bundle was employed to study ischemia-induced cell mitosis in the adult rat retina [53]. The model by ligation of ophthalmic vessels (LOV) was applied to reveal ischemia-induced retinal ganglion cell death [54]. Damages to the retina in these models may be more serious than purely ischemia injury because the mechanical injury may also contribute to the retinal damage. The ischemia model by occlusion of the bilateral common carotid arteries (BCCAO) has also been employed to investigate the pathological changes of the retina in several studies [29], [30], [41]. All authors found a loss of retinal ganglion cells after BCCAO but due to variable chronic occlusion times they reported different latencies of ganglion cell death. The earliest time point for ganglion cell loss was 7 days after the start of occlusion as found by Yamamoto et al. The 4-VO ischemia model used in the present study produces quite different results from the BCCAO model. After approximately 17 min of complete occlusion followed by reperfusion we did not find any ganglion cell loss but a loss of immunoreactivity by some ganglion cells (and amacrine cells). Compared to the retinal ischemic models mentioned above, the 4-VO model has been employed less to observe the changes in pathology of the retina. Ozden, S. et al reported that transient global ischemia for a duration of 30 to 90 min induced the loss of ganglion cells and amacrine cells [55]. Our study showed that an approximate 17 min duration of transient global ischemia could induce changes in ERG recording and synthesis of protein of ganglion cells and amacrine cells, and anxiety-like behavior but not cell loss.

In conclusion, transient global ischemia for 17 min induced cellular, molecular and electrophysiological dysfunction of RGCs and amacrine cells within 2 days after ischemia, but did not affect photoreceptors and light perception. Bipolar cells underwent transient changes in molecular and electrophysiological levels after ischemia. The results support the idea that RGCs and amacrine cells are most vulnerable and photoreceptors are most resistant to ischemia.
